# Hyperactivation of PARP Triggers Nonhomologous End-Joining in Repair-Deficient Mouse Fibroblasts

**DOI:** 10.1371/journal.pone.0049301

**Published:** 2012-11-07

**Authors:** Natalie R. Gassman, Donna F. Stefanick, Padmini S. Kedar, Julie K. Horton, Samuel H. Wilson

**Affiliations:** Laboratory of Structural Biology, National Institute of Environmental Health Sciences, Research Triangle Park, North Carolina, United States of America; King Faisal Specialist Hospital & Research Center, Saudi Arabia

## Abstract

Regulation of poly(ADP-ribose) (PAR) synthesis and turnover is critical to determining cell fate after genotoxic stress. Hyperactivation of PAR synthesis by poly(ADP-ribose) polymerase-1 (PARP-1) occurs when cells deficient in DNA repair are exposed to genotoxic agents; however, the function of this hyperactivation has not been adequately explained. Here, we examine PAR synthesis in mouse fibroblasts deficient in the base excision repair enzyme DNA polymerase β (pol β). The extent and duration of PARP-1 activation was measured after exposure to either the DNA alkylating agent, methyl methanesulfonate (MMS), or to low energy laser-induced DNA damage. There was strong DNA damage-induced hyperactivation of PARP-1 in pol β nullcells, but not in wild-type cells. In the case of MMS treatment, PAR synthesis did not lead to cell death in the pol β null cells, but instead resulted in increased PARylation of the nonhomologous end-joining (NHEJ) protein Ku70 and increased association of Ku70 with PARP-1. Inhibition of the NHEJ factor DNA-PK, under conditions of MMS-induced PARP-1 hyperactivation, enhanced necrotic cell death. These data suggest that PARP-1 hyperactivation is a protective mechanism triggering the classical-NHEJ DNA repair pathway when the primary alkylated base damage repair pathway is compromised.

## Introduction

Poly(ADP-ribose) polymerase-1 (PARP-1) is an abundant nuclear protein with functions linked to transcription, chromatin remodeling, replication, and both single-strand break (SSB) and double-strand break (DSB) DNA repair. These diverse functions have confounded efforts toward determining the exact roles of PARP-1 in DNA repair pathways, including base excision repair (BER). PARP-1 binds rapidly to strand break-containing repair intermediates and catalyzes polymerization of ADP-ribose moieties from nicotinamide adenine dinucleotide (NAD^+^) onto itself and other nuclear proteins (termed PARylation). PARP-1 accounts for most of the PARylation in the cell. Synthesis of these poly(ADP-ribose) polymers results in the recruitment of BER proteins, e.g., XRCC1, DNA ligase III, and DNA polymerase β (pol β) to sites of DNA damage [1], [2], [3]. PARylation is critical to the process of BER/SSB repair. Inhibition of PARP activity has been shown to impair recruitment of BER proteins [1], [2], [3], [4] and to increase levels of cytotoxic BER intermediates [5]. Cell death results either through accumulation of toxic DNA intermediates [5], [6] or through replication fork collapse and replication-dependent DSB formation [7], [8], [9]. Along with the detrimental effects observed with the loss of PARylation through PARP inhibition, hyperactivation of poly(ADP-ribose) (PAR) synthesis by PARP-1 can deplete intracellular ATP levels [10], [11]. Furthermore, the PAR polymer itself can be toxic since it acts as a cell death effector resulting in PAR-mediated cell death, also known as parthanatos [12], [13].

With the extremes of too little and too much PAR known to result in cell death, the ability of cells to produce the appropriate amount of PAR for the recruitment of DNA repair proteins is critical to the regulation of repair required following DNA damage. Recently, elevated PAR levels were observed when deficiencies in the BER protein, pol β [14], or the homologous recombination (HR) protein, BRCA2 [15] occurred. In repair-deficient cell lines, PAR levels were slightly elevated over wild-type cells in the absence of DNA damage, and hyperactivation of PARP-1 occurred after treatment with DNA damaging agents. However, the function of this hyperactivation of PAR synthesis has not been adequately explained. In the present study, our initial evaluation of MMS-treated mouse fibroblasts revealed hyperactivation of PAR synthesis in BER-deficient cells, but this did not lead to cell death, as previously suggested [13], [14]. Previous studies have proposed that PARP-1 acts as a sensor at DNA damage sites [16], [17], and here we demonstrate that the PARP-1 sensor overproduces PAR when deficiencies in BER exist. We propose PAR overproduction signals damage site recruitment of nonhomologous end-joining (NHEJ) factors, enabling a backup repair pathway. To test this hypothesis, we investigated the extent, duration, and protein-interactions associated with PARP-1 activation in response to DNA damage in wild-type cells or those deficient in pol β.

## Materials and Methods

### Cell Culture

Wild-type and pol β null SV40-transformed mouse embryonic fibroblasts (MB36.3 and MB38Δ4, referred to as wild-type and pol β null cells, have been described previously [18]. Cells were routinely grown at 34°C in a 10% CO_2_ incubator in Dulbecco's modified Eagle's medium (DMEM) supplemented with GlutaMAX-1 (Life Technologies, Carlsbad, CA), 10% fetal bovine serum (FBS; HyClone, Logan, UT), and hygromycin (80 µg/ml; Roche Molecular Biochemicals, Indianapolis, IN). Cells were routinely tested and found to be free of mycoplasma contamination.

### Western blotting and co-immunoprecipitation

Wild-type and pol β null cells were seeded in 145 mm dishes at 10^6^ cells/dish and treated when cells were 90% confluent with 10 mM MMS (Sigma-Aldrich, St. Louis, MO) at 4°C in growth medium without hygromycin but supplemented with 25 mM HEPES. After a 20 min exposure, cells were washed in ice-cold Hanks' balanced salt solution (HBSS, HyClone) and fresh, warm growth medium was added. Dishes were incubated for the indicated time at 34°C in a 10% CO_2_ incubator, then immediately placed on ice. Cells (either treated as described, or control, untreated) were washed in phosphate buffered saline (PBS) and collected by scraping, suspended in two volumes of lysis buffer (50 mM Tris-HCl, pH 7.5, 150 mM NaCl, 25 mM NaF, 0.1 mM sodium orthovanadate, 0.2% Triton X-100, and 0.3% NP-40) containing protease inhibitors, 0.1 mM PMSF, 1 µg/ml aprotinin, and 5 µg/ml leupeptin [19] and incubated on ice for 30 min. After agitating the tubes briefly, the lysates were centrifuged at 20,800×g for 30 min at 4°C, and the supernatant fraction was removed. Protein concentrations were determined using the Bio-Rad protein assay with bovine serum albumin (BSA) as standard.

For PAR immunodetection, 30 µg of prepared lysates from control and treated cells were separated by 8% SDS-PAGE. The proteins were then transferred onto a nitrocellulose membrane in a transblot apparatus for 2 h at 150 mA. The membrane was blocked with 5% nonfat dry milk in Tris-buffered saline (TBS) containing 0.1% (v/v) Tween-20 (TBS-T) and probed with the anti-PAR monoclonal antibody (1∶1,000 dilution; Trevigen, Gaithersburg, MD). Detection was by ECL following incubation with secondary antibody conjugated to HRP. The blot was stripped by incubating with buffer containing 62.5 mM Tris-HCl, pH 6.8, 100 mM β-mercaptoethanol, and 1% SDS for 30 min at 50°C, or with Restore Western Blot Stripping Buffer as suggested by the manufacturer (Pierce Biotechnology, Inc.), then washed twice for 30 min with room temperature TBS-T. After stripping, the membrane was then probed with anti-PARP-1 monoclonal antibody (1∶1,000; BD Biosciences, San Jose, CA), and then with anti-Tubulin (1∶10,000; Sigma-Aldrich) as a loading control.

For co-immunoprecipitations with PARP-1, an equal amount (1 mg protein) of each cell extract was mixed with 1–2 µg of anti-PARP-1 rabbit polyclonal antibody (Enzo Life Sciences, Farmingdale, NY). The mixture was incubated with rotation for 4 h at 4°C. The immunocomplexes were adsorbed onto protein A-sepharose and protein G-agarose beads (1∶1 mixture) by incubating the mixture for 16 h at 4°C. The beads were then washed four times with lysis buffer containing protease inhibitors. Finally, the beads were resuspended in SDS sample buffer, heated at 95°C for 5 min, and briefly centrifuged. The soluble proteins were separated by electrophoresis on 4–12% SDS-PAGE in MOPS buffer and then transferred to a nitrocellulose membrane for 2 h. The membrane was blocked and first probed with anti-Ku70 monoclonal antibody (1∶300 dilution; Santa Cruz Biosciences), then anti-PAR antibody (1∶1000), and finally, anti-PARP-1 monoclonal antibody (1∶1,000). In control experiments, the immunoprecipitating antibody was substituted with normal goat or rabbit agarose-conjugated IgG. Cell extract (30 µg) without immunoprecipitation was also used as a source of marker proteins (Input) and was subjected directly to SDS-PAGE and immunoblotting as presented in the figures.

### Determination of total cellular PAR

Cellular PAR levels were quantified using the PARP *in vivo* Pharmacodynamic Assay 2nd Generation (PDA II) kit (4520-096-K, Trevigen) following the kit protocol and treatment protocol described above. Briefly, wild-type and pol β null cells were seeded in 60 mm dishes at 10^6^ cells/dish and treated the next day with 10 mM MMS at 4°C in growth medium without hygromycin but supplemented with 25 mM HEPES. After 20 min exposure, they were washed in ice-cold HBSS and fresh, warm growth medium was added. Dishes were incubated for the indicated time at 34°C in a 10% CO_2_ incubator, then immediately placed on ice and lysed according to the kit protocol. Control cells were treated similarly and collected after 10 min. Cell counts for both wild-type and pol β null cells were verified to ensure no growth differential between cell lines. After cell lysis and DNA digestion, total protein amounts were determined for each sample and 10 µg of wild-type or 4 µg of pol β null cell extract were added to pre-coated capture antibody plates and incubated overnight at 4°C. The following morning, wells were washed four times in 1× PBS with Tween-20 (PBST), and then 1∶250 dilution of PAR polyclonal detecting antibody was added and incubated at room temperature for 2 h. Wells were again washed four times in 1× PBST, then 1∶250 dilution of goat anti-rabbit IgG-HRP was added and incubated for 1 h. Cells were washed again four times in 1× PBST, then a 1∶1 mixture of PARP PeroxyGlow™ A and B was added and luminescence was measured with a Tropix TR717 Microplate Luminometer. For treatment with 4-amino-1,8-naphthalimide (4-AN, Sigma-Aldrich) and pamoic acid (PA, Sigma-Aldrich), the cell medium was removed approximately 6 h after plating, and growth medium with 10 µM 4-AN or 300 µM PA was added, and cells were further incubated for sixteen hours. These concentrations of inhibitor were maintained during treatment with MMS and during the repair times.

### Micro-irradiation and immunofluorescence

Wild-type and pol β null cells were seeded on 35 mm glass bottomed petri dishes containing an etched grid (MatTek, Ashland, MA) at 2×10^5^ cells per dish and incubated in growth medium without hygromycin and supplemented with 10 µM BrdU (Sigma-Aldrich) for 24 h. After 24 h, medium was exchanged to medium without hygromycin and BrdU. Samples were then imaged using a 40× C-Apochromat (numerical aperture 1.2) water immersion objective coupled to a Zeiss LSM510 META confocal microscope (Carl Zeiss MicroImaging). Base lesions and strand breaks were introduced by UV laser micro-irradiation at 364 nm (Coherent Enterprise II) with intensities equivalent to 0.176 µJ. This energy requirement was previously characterized by Lan et. al. [2] to induce the recruitment of BER proteins without producing DSBs, as determined by the presence of γH2AX at damage sites. To replicate these conditions, we measured the energy per pixel output of the UV laser coupled to the LSM with an Xcite XR2100 power meter with a XP750 objective plane power sensor (Lumen Dynamics, Mississauga, Ontario, CA). We then determined the number of scanning iterations (∼200) required to deliver 0.176 µJ over a fixed pixel length. To verify we were producing primarily base lesions and SSB breaks and not DSB breaks, we examined the recruitment of XRCC1 to sites of damage as shown below and in a prior publication [20], and found we were consistent with previously published studies [2], [3]. Additionally, we assayed the production of DSBs at this energy was using anti-γH2AX monoclonal antibody (Millipore, Billerica, MA), and similar to Lan et. al., [2] no γH2AX foci were observed (data not shown).

After micro-irradiation, cells were either immediately fixed in 4% paraformaldehyde or allowed to recover in a 34°C incubator for the times indicated. After fixation, cells were permeabilized with 0.25% Triton X-100 in PBS for 10 min, washed three times in PBS, then further permeabilized and blocked with PBS+1% BSA for 30 min. Cells were then incubated with anti-XRCC1 antibody (1∶50; Abcam, Cambridge, MA) and anti-PADPR antibody (1∶100; Abcam) for 1 h. Cells were washed three times with PBS, then incubated in Alexa 488 conjugated anti-mouse and Alexa 647 conjugated anti-chicken (1∶2,000; Life Technologies) for 1 h. For pol β imaging, cells were permeablized in 1% SDS as previously described [21], and then incubated with anti-pol β antibody (1∶200, Abcam) for 1 h. Cells were washed three times with PBS, then incubated in Alexa 546 conjugated anti-rabbit (1∶2,000; Life Technologies). Fluorescence images were acquired with the same 40× water immersion objective on the LSM510. Recruitment of XRCC1 and pol β, and synthesis of PAR at the site of DNA damage was measured using IMAGEJ. The mean intensity of the irradiated site was determined by measuring the intensity of the fluorescence along the irradiation line (I_UV_) and dividing that value by the intensity of the fluorescence in the same nucleus away from the site of DNA damage (I_N_). Both the I_UV_ and I_N_ values were background subtracted and normalized to line length to account for differences in nucleus size. Using this procedure, the mean intensity in control cells (not irradiated) was determined and a resulting value of ∼1 was determined for all measurements. This value was subtracted from the irradiated (I_UV_/I_N_) ratio to reflect the increase in mean intensity observed after irradiation. Values of 0 for mean intensity reflect equal distribution of proteins thoughout the nucleus, while values above 0 reflect an increasing protein concentration along the site of DNA damage. Each experiment was repeated on at least five cells, and the data presented here represent mean values. Images are representative.

### Intracellular ATP assay

Intracellular ATP levels were measured as previously described [10]. Briefly, wild-type and pol β null cells were seeded in 145 mm dishes at 6.9×10^5^ cells per dish. The following day, cells were treated with MMS as described above. Cells were harvested by scraping after the appropriate repair time and suspended in PBS. After counting, cells (10,000 per well) were added to opaque-walled 96-well dishes in culture medium. The cells were then lysed by addition of CellTiter-Glo™ reagent (Promega, Madison, WI), and ATP levels were determined by measuring luminescence with a Tropix TR717 Microplate Luminometer.

### Analysis of cell death by flow cytometry

Wild-type and pol β null cells were seeded in 100 mm dishes at 3×10^5^ cells/dish and treated as described. DNA-PK inhibitor NU-7026 was incubated with cells during the MMS exposure, and then the incubation was continued until harvesting for analysis. Stock solutions of NU-7026 were made in DMSO and stored at −20°C; the final concentration in medium was 15 µM. At 24 h after MMS exposure, the cells were harvested using 0.05% trypsin and collected with the culture medium. After centrifuging and washing with PBS, annexin V-fluorescein isothiocyanate (FITC) and propidium iodide (PI) were added in binding buffer as suggested by the manufacturer (TACS™ Annexin V Apoptosis Detection Kit, Trevigen). The samples were incubated at room temperature in the dark for 15 min, read on a FACS flow cytometer, and analyzed using Cell Quest software (Becton-Dickinson Immunocytometry Systems, San Jose, CA). Data were analyzed as previously described [10].

## Results

### Deficiency in pol β results in hyperactivation of PAR synthesis following alkylating damage in mouse fibroblasts

**Figure 1:**
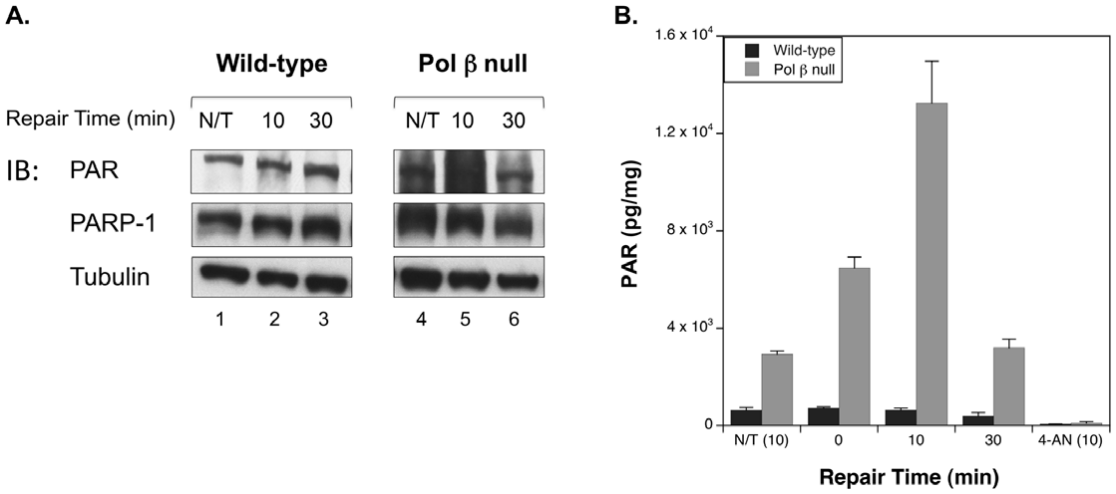
PARP-1 is hyperactivated following MMS treatment of pol β null cells. A. Immunoblot of PAR in untreated cells (N/T) and wild-type and pol β null cells treated with 10 mM MMS for 20 min at 4°C, then incubated without MMS at 34°C for the repair time specified. B. Time course of total cellular PAR levels of untreated (N/T (10), on graph) cells and cells treated with MMS or MMS+10 µM 4-AN (4-AN (10), on graph). Samples treated with 4-AN were pretreated at 34°C for 16 h prior to MMS exposure. 10 mM MMS was added in the presence of 4-AN for 20 min at 4°C, then the MMS was removed and cells were allowed to repair for 10 min in the presence of 4-AN at 34°C. Each experiment was repeated at least three times and error bars reflect SEM.

We initially determined the level of PAR synthesis or PARylation in wild-type and pol β null cells in response to alkylating agent-induced DNA damage. Cells were exposed for 20 min to a high dose of MMS (10 mM) in the cold. These treatment conditions were previously characterized by the alkaline comet assay [22] and allow DNA damage to accumulate rapidly in the cells, but with minimal DNA repair. In initial experiments using SDS-PAGE of cell extracts and immunoblotting, an elevated level of PARylation was observed at 10 min (Figure 1A, lane 5) in extracts from pol β null cells compared with wild-type cells (lane 2), and this elevated PARylation state decreased within 30 min of exposure to approximately the untreated cell levels (lane 6). While PAR synthesis can be monitored by immunoblotting, quantifying total intracellular PAR provides a more accurate assessment of the PARylation status after MMS treatment. As shown in Figure 1B, an elevation in PAR level occurred immediately after MMS exposure (0 min) in the pol β null cells compared with untreated (N/T) cells, and continued until it reached a maximal state after 10 min repair. After this time, the PAR level decreased rapidly, returning to the untreated level at 30 min (Figure 1B) and 1 h post treatment (not shown). Wild-type cells failed to show a significant fluctuation in PAR level after MMS exposure, maintaining a relatively steady-state level of PAR, presumably as a result of efficient DNA repair [22]. Addition of the PARP inhibitor 4-AN during the MMS-induced damage to cells eliminated the hyper-PARylation, as expected (Figure 1B).

**Figure 2:**
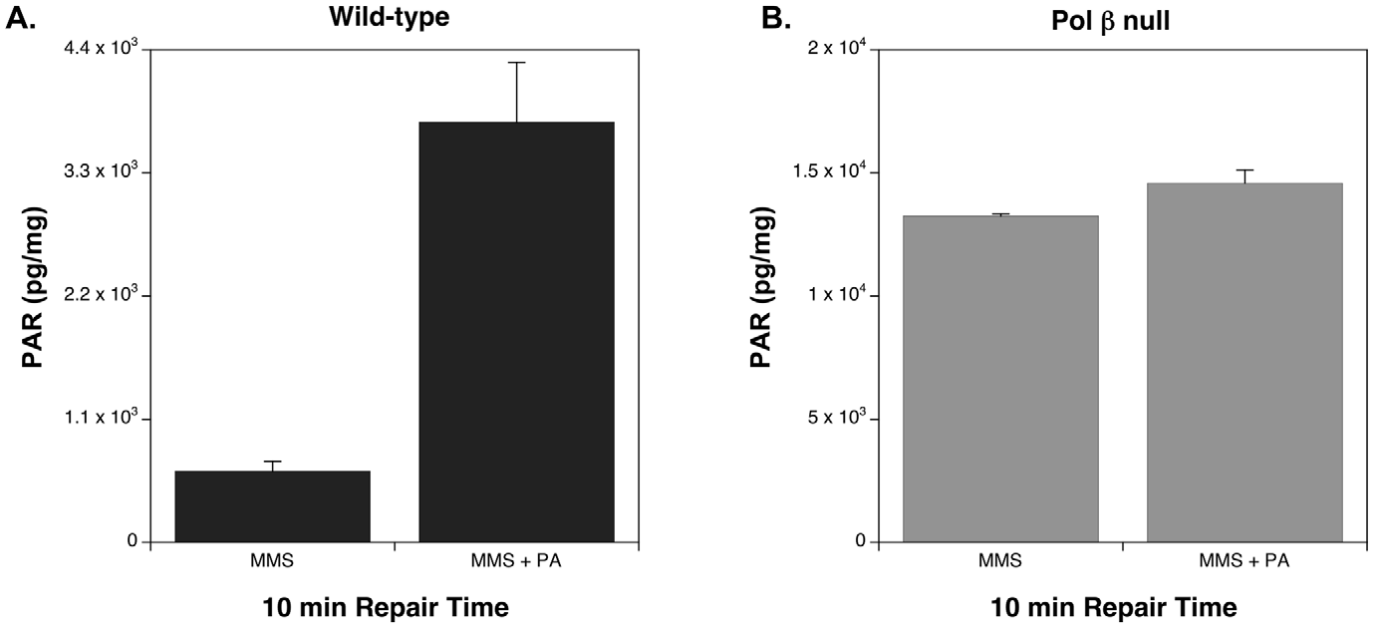
Total cellular PAR levels of cells treated with MMS and MMS+300 µM PA (PA). A. Wild-type cells treated with MMS and MMS ±300 µM PA (PA). Samples treated with PA were pretreated at 34°C for 16 h prior to MMS exposure. 10 mM MMS was added in the presence of PA for 20 min at 4°C, then the MMS was removed and cells were allowed to repair for 10 min in the presence of PA at 34°C. B. Pol β null cells treated as described in A. Each experiment was repeated at least three times and error bars reflect SEM.

To further test that deficient BER results in the hyperactivation of PARP-1, we treated both wild-type and pol β null cells with the most active and specific pol β inhibitor available, pamoic acid (PA) [23]. Addition of PA to MMS-treated wild-type cells resulted in a significant increase in PARylation 10 min after MMS exposure (Figure 2A), though not to the maximal level observed in MMS-treated null cells. As expected, incubation with PA had a negligible effect in pol β null cells (Figure 2B).

### Deficiency in pol β results in hyperactivation of PARP following low energy laser-induced DNA damage

**Figure 3:**
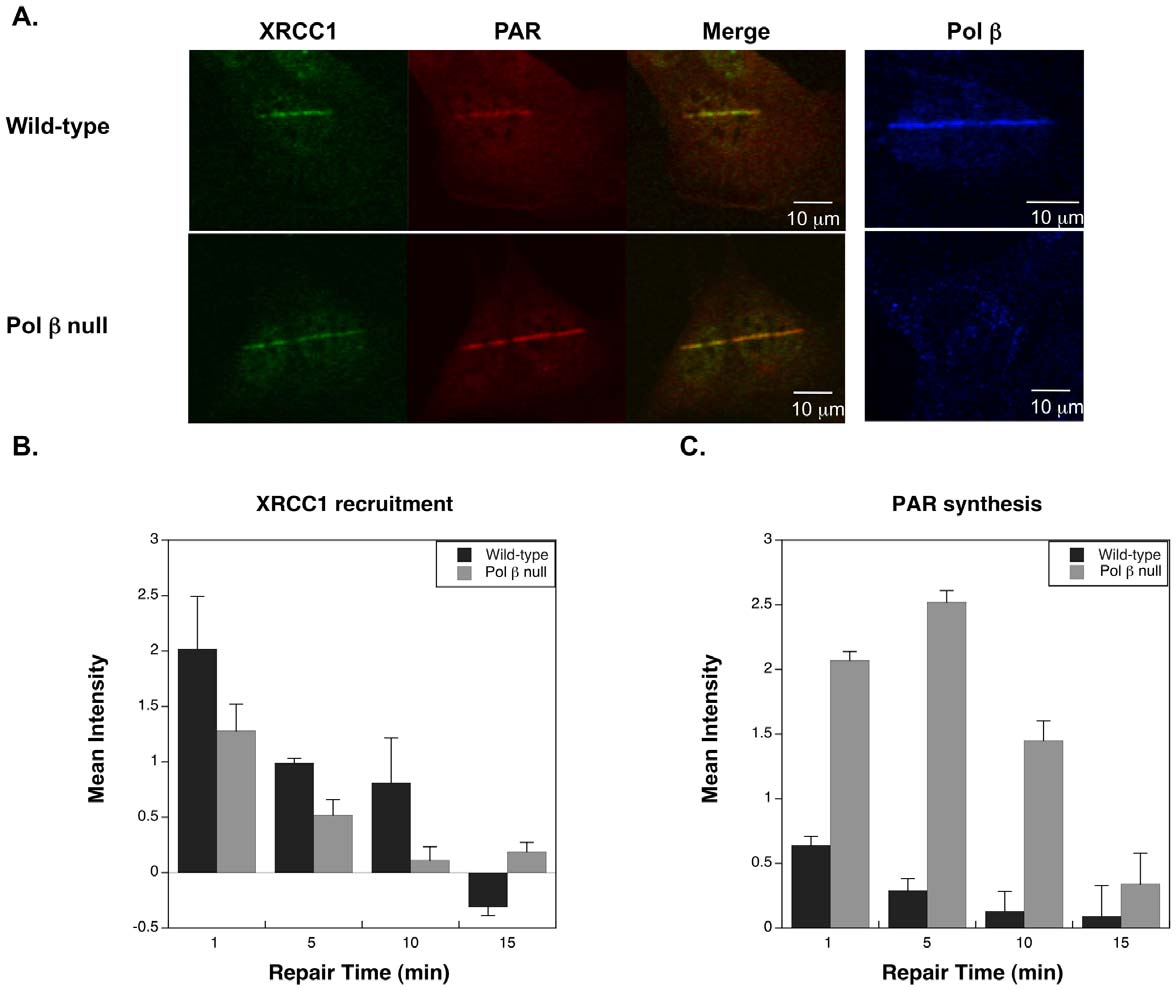
PARP-1 hyperactivation in pol β null cells in response to UV damage. A. Recruitment of BER proteins as indicated to the site of DNA damage in wild-type and pol β null cells one minute after irradiation. B. Time course of XRCC1 recruitment to the site of damage with the repair times specified. C. PAR synthesis at the site of damage with the repair times specified. Images are representative; at least five cells were measured for every time point and error bars reflect SEM.

Micro-irradiation studies had previously demonstrated recruitment of BER proteins to sites of laser delivered UV-induced DNA damage in cells [2], [3], [4]. Using low laser power (∼0.17 µJ), base lesions and SSBs are preferentially made over DSBs [2], and this was verified here by the absence of γH2AX staining with the exposure conditions used (not shown). Under these conditions, recruitment of XRCC1 and synthesis of PAR were observed within a minute of irradiation in both cell lines (Figure 3A). Pol β also was recruited under these conditions in the wild-type cells (Figure 3A, right panel). Cells transfected with GFP-PARP-1 showed PARP-1 recruitment to the sites of damage (not shown), but we were unable to optimize the immunofluorescence conditions to enable imaging of endogenous PARP-1 protein.

In wild-type cells, XRCC1 protein and PAR synthesis was detectable within 30 sec of irradiation, and reached a maximum at 1 min post-irradiation (Figure 3B and C). In pol β null cells, XRCC1 recruitment was on the same time scale as in the wild-type cells; however, the recruitment was slightly less robust. Additionally, and in agreement with the results presented in Figure 1, quantification of PAR synthesized at sites of damage demonstrated considerable hyperactivation of PARP-1 in the null cells (Figure 3C). This increased PARylation in the null cells reached a maximum at 5 min post-irradiation and continued until 15 min post-irradiation.

**Figure 4:**
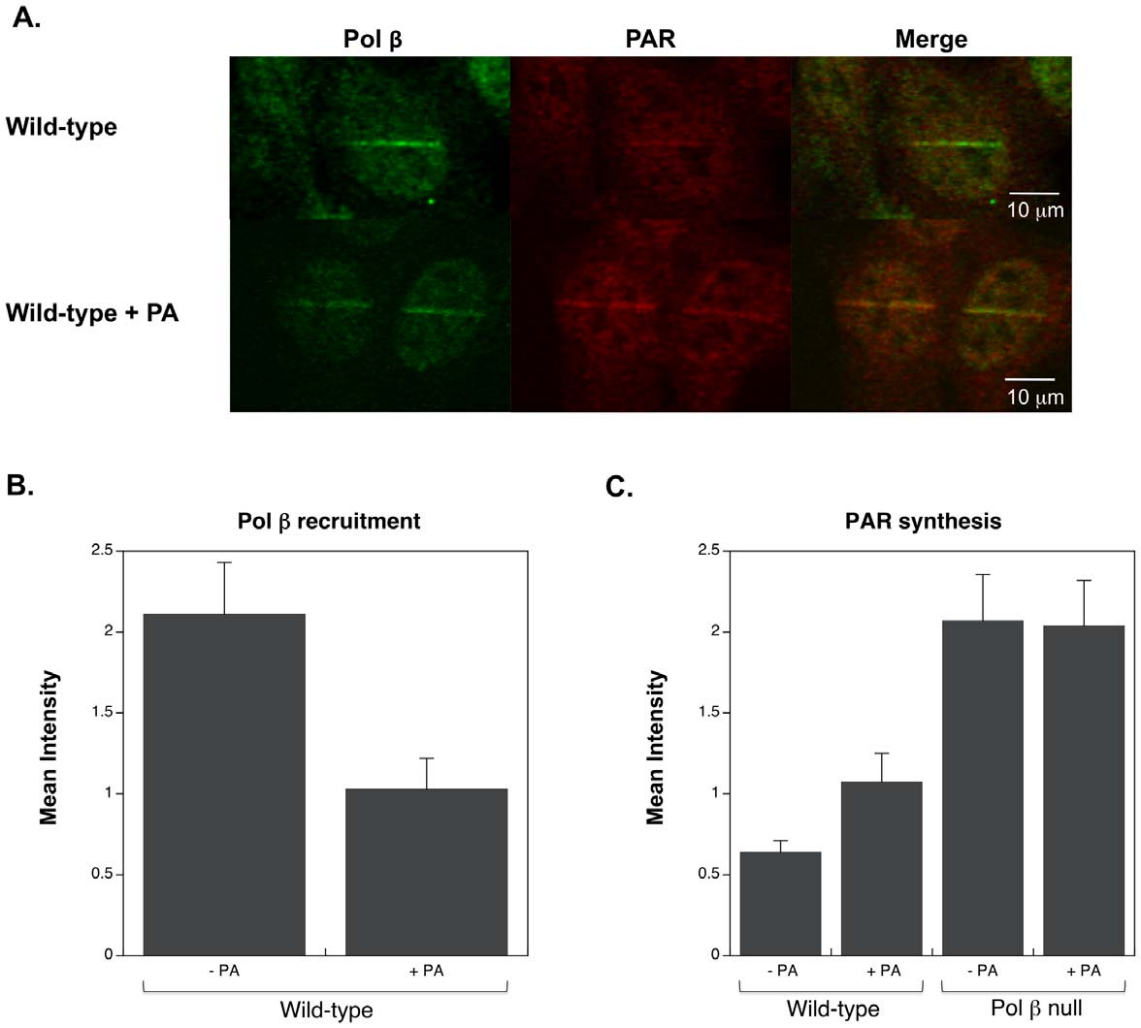
Effect of PA on recruitment of pol β and PAR synthesis. A. Images one minute after irradiation in wild-type cells, and wild-type cells treated with 300 µM PA. B. Pol β recruitment one minute after irradiation in the absence and presence of PA. C. PAR synthesis at the site of damage one minute after irradiation in the absence and presence of PA. Images are representative; at least five cells were measured for every time point and error bars reflect SEM.

Incubation of wild-type cells with the pol βinhibitor PA reduced the recruitment of pol β to the sites of damage, as shown in Figure 4A and quantified in Figure 4B. However, PA treatment did not completely eliminate pol β recruitment. This deficiency in pol β recruitment correlated with an increase in PAR synthesis at the damage sites, although not to the degree observed in the pol β null cells (Figure 4C).

### PARP-1 hyperactivation and cell death

Hyperactivation of PARP-1 has been postulated to initiate necrosis through a massive depletion of NAD^+^ [24], [25], [26]. Depletion of intracellular NAD^+^ by increased synthesis of PAR polymers could result in depletion of intracellular ATP levels leading to necrosis [25], [27]. To determine the influence of MMS dosing and PARP-1 hyperactivation in the present work, intracellular ATP levels were measured at times up to 24 h after MMS exposure and no significant depletion was observed (Figure S1). Therefore, the treatment conditions utilized in this current study resulted in hyperactivation of PARP-1 but not depletion of ATP.

In addition to energy depletion, the PAR polymer has been shown to act as a cell death effector [13], [28]. To exclude cell death through parthanatos, cells were analyzed for apoptotic and necrotic cell death using annexin and PI staining. Only a small fraction of cells were undergoing either apoptosis (wild-type 2.1±0.4% and pol β null 2.9±0.7%) or necrosis (1±0.7% and 5.4±0.7%) 24 h after treatment with MMS (data not shown). Therefore, the elevated levels of PAR observed in both untreated and MMS-treated pol β null cells were unable to deplete ATP or to initiate necrosis.

### Hyperactivation of PARP-1 increases interaction with Ku70

We considered that the excess PAR generated in MMS-treated pol β null cells serves as a signal for recruitment of a protective backup DNA repair system at the sites of damage. It had previously been demonstrated that PAR is critical to damage site recruitment of DSB repair proteins [1], [2], [3], [4], [29], and PAR synthesis also is known to be stimulated by DSBs *in vitro* and *in vivo* [27], [30], [31]. In addition, PARP-1 was shown to be critical for the alternate or backup-NHEJ pathway [32], [33], [34], [35], and a number of proteins involved in classical-NHEJ are known to interact with PAR through a pADPr (poly(ADP-ribose)) binding motif [36], [37] or to be targets of PARylation by PARP-1 [27], [38], [39].

**Figure 5:**
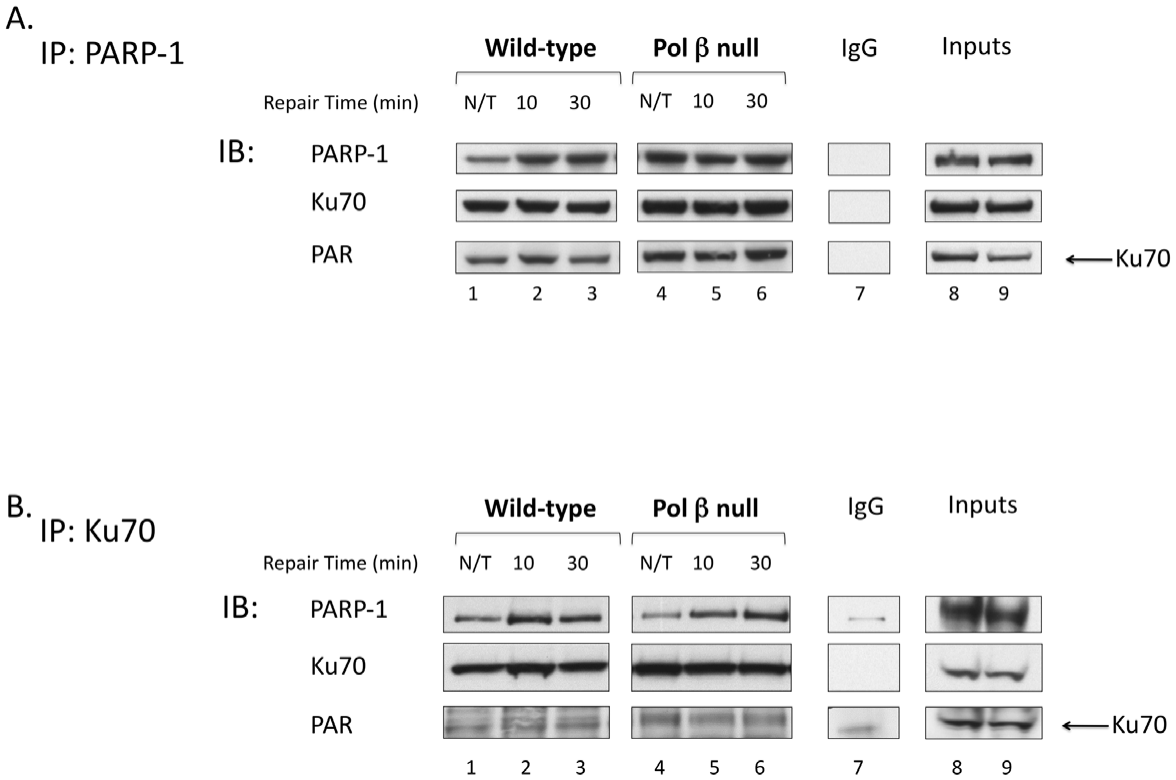
Interaction of PARP-1 and Ku70 after MMS exposure. A. Immunoprecipitation with anti-PARP-1 antibody from untreated (N/T) and MMS-treated cell extracts with the repair times specified. B. Immunoprecipitation with anti-Ku70 antibody as in A. Immunoblotting (IB) was performed with the antibodies specified. An IgG negative control was performed for both immunoprecipitations (lanes 7), and 30 µg of cell extract (input wild-type and pol β null, respectively) was used as a source of marker proteins (lanes 8 and 9).

To determine if the excess PAR generated in the pol β null cells resulted in increased interaction with a DSB repair protein, we examined the interaction of Ku70 with PARP-1. Ku70 can be PARylated by PARP-1 [38] and binds to PAR through a well described pADPr motif [36], [37]. It is also known that Ku70 can compete with the backup-NHEJ pathway and stimulate classical-NHEJ [33], [34], [40], [41]. Typical results of the immunoprecipitation with anti-PARP-1 antibody are shown in Figure 5A. With wild-type cells, co-immunoprecipitation was observed between PARP-1 and Ku70 for both MMS treated and untreated cells (lanes 1–3), and with pol β null cells, Ku70 immunoprecipitation was stronger after MMS treatment (lanes 4–6). Immunoblotting with anti-PAR antibody revealed PAR adduction of Ku70 in all of the samples (bottom panel). Immunoprecipitation with anti-Ku70 antibody also was conducted (Figure 5B). With both wild-type and pol β null cells, more PARP-1 was immunoprecipitated after MMS treatment (top panel). An IgG control was performed for both immunoprecipitations and no significant association was noted with any of the proteins identified (Figure 5A and B, lane 7).

### Inhibition of DNA-PK induces necrotic cell death in MMS treated pol β null cells

The association of PARP-1 and Ku70 in MMS-treated pol β null cells suggested a backup role for NHEJ in cellular protection against MMS in BER-deficient cells. Classical-NHEJ is executed by the DNA-dependent protein kinase (DNA-PK) holoenzyme, a complex composed of the Ku70/Ku80 heterodimer together with the catalytic subunit of DNA-PK (DNA-PKcs), and a complex of XRCC4, DNA ligase IV, Artemis, and XLF/Cernunnos [42], [43], [44]. Previous studies demonstrated that NU-7026 (2-(morpholin-4-yl)-benzo[h]chomen-4-one) is a potent inhibitor of both DNA-PK and NHEJ [45], but does not affect the backup-NHEJ pathway which utilizes PARP-1, XRCC1, and DNA ligase III [33].

**Figure 6:**
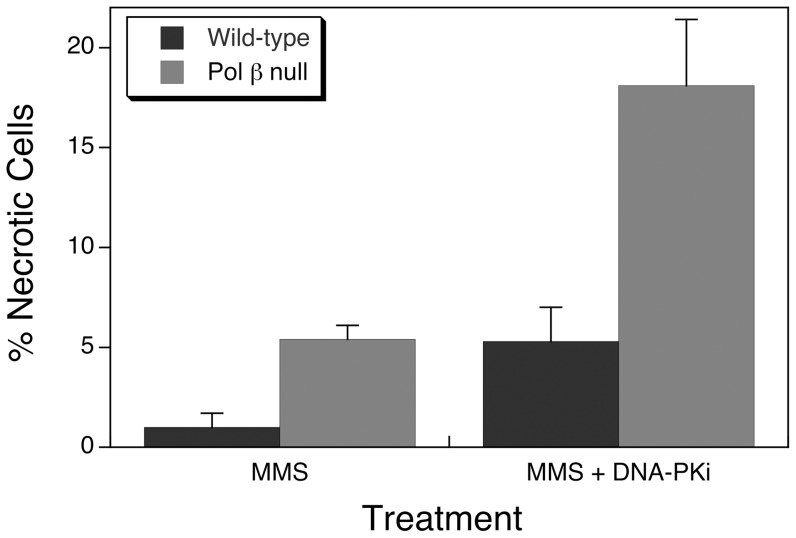
Necrotic cells measured by flow cytometry 24 h after treatment. Cells were treated with 10 mM MMS for 20 min at 4°C, then cells were washed and incubated at 34°C until harvested for analysis. The DNA-PK inhibitor, NU-7026 was co-treated with MMS for 20 min at 4°C, then the MMS was removed and cells were incubated at 34°C in the presence of NU-7026 until harvested for analysis (MMS+DNAPKi). Values represent the means ± SEM of three independent experiments normalized to the control experiment.

Cells were examined by flow cytometry after treatment with MMS alone or in combination with NU-7026. Annexin and PI staining revealed only small increases in the proportion of apoptotic cells (wild-type 5.2±1.4% and pol β null 5.2±0.9%) with combined MMS and NU-7026 treatment, while there was a significant increase in necrotic cell death in pol β null cells after the combined treatment (Figure 6). Treatment with NU-7026 alone had only a small effect in wild-type cells and a negligible effect of pol β null cells (not shown). Together, these results suggest a role for DNA-PK-dependent NHEJ in the survival of pol β null cells after MMS-induced damage.

## Discussion

PARP-1 is an abundant nuclear protein with diverse and wide-ranging functions in the cell. The ubiquitous presence of PARP-1 across various processes in DNA metabolism has complicated understanding the unique roles it plays in BER/SSB and DSB repair. Despite the ambiguity in its functions, it is clear that PARP-1 activation is important in the cellular response to DNA damage. PAR synthesis directly impacts protein recruitment to sites of DNA damage, it can regulate the efficiency of repair, and in some cases can even lead to cell death.

Here, we examined PAR levels associated with BER-proficiency (wild-type) and BER-deficiency (pol β null) in mouse fibroblast cells. The PAR level increased dramatically in pol β null cells after exposure to the DNA alkylating agent, MMS (Figure 1) and also after laser irradiation of cells in cell imaging experiments (Figure 3). Using this micro-irradiation and imaging approach, we showed that the pol β inhibitor PA partially blocked pol β from being recruited to the sites of DNA damage; therefore, the increase in PAR associated with the presence of PA appeared to reflect a defect in pol β recruitment. From these data, we proposed that the elevated PAR level may act as a reporter of a deficiency in DNA repair. Consistent with this hypothesis, a study by Gottipati et. al., found that deficiencies in BRCA2 or shRNA depletion of *RAD54, RAD52, BLM, WRN, and XRCC3* all resulted in hyperactivation of PARP-1 [15]. Thus, PARylation may play a role in the amplification of DNA damage and repair signaling. Since the PARP-1 hyperactivation state in pol β null cells is transient and does not induce necrosis, it is probable that the role of the increased PARylation is to signal a deficiency in BER. In the absence of such a deficiency, there was no increase in PARylation after MMS treatment (Figure 1). In addition to hyper-PARylation serving as an indicator for deficiency in BER, recruitment of DNA repair proteins by PAR has been well documented [1], [3], [4], [46]. We propose that hyperactivation of PARP-1 serves as the signal for recruitment of a back-up DNA repair system to the sites of damage, allowing for repair and increased cell viability in the absence of pol β.

PARP-1 is critical to DSB repair by the backup-NHEJ pathway. This backup pathway, involving PARP-1, XRCC1, and DNA ligase III, in concert with MRE11 and NBS1 (MRN) and possibly other as yet unidentified proteins, is responsible for the residual end-joining of DSBs in cells deficient in components of classical NHEJ and is independent of Ku70/Ku80 and DNA-PK [33], [34], [35], [41], [42]. While the components and mechanisms of this pathway are still under study, it has been established that the kinetics of DSB repair are slower than classical-NHEJ [41] and that it is repressed by Ku under normal conditions [47], [48], [49], [50]. Examination of the interaction of PARP-1 with Ku70 revealed increased association after MMS treatment, and an increased association of PAR-adducted Ku70 with PARP-1 in pol β null cells (Figure 5). With this emerging picture about the relationship between PARP-1, PARylation, and Ku70, we aimed to verify a protective role of NHEJ in pol β null cells by utilizing the DNA-PK inhibitor, NU-7026, in combination with MMS treatment. Under these DNA-PK inhibited conditions, there was enhanced necrotic cell death of pol β null cells compared with wild-type cells. This increase suggested a link between PARP-1 hyperactivation and DNA-PK-dependent NHEJ in MMS-treated pol β-deficient cells.

During NHEJ, damaged DNA ends are captured by the Ku heterodimer (Ku70/Ku80) that recruits and activates DNA-PKcs, which in turn mediates the ligation of the DNA ends by the DNA ligase IV/XRCC4/XLF complex [42], [43], [44]. Ku70 and DNA-PK together compose the key complex in classical-NHEJ. Ku70 competes for DNA binding with PARP-1 making classical-NHEJ the dominant pathway over PARP-1-dependent backup-NHEJ [34], [35], [41]. *In vitro* PARylation of Ku70 was shown to reduce its DNA-binding activity and to inhibit classical-NHEJ [38]. In addition to covalent modification by PAR, Ku70 has a pADPr binding motif (a.a. 246–261) that may enhance its interaction with PARylated proteins [36], [37]. PARylation of the DNA-PK catalytic subunit by PARP-1 has been shown to enhance its kinase activity [51], [52], supporting its stimulatory role in DSB repair [33]. In addition, a recent study in *Dictyostelium discoideum* demonstrated that the Ku70 PAR-binding zinc finger (PBZ) [53] is required for recruitment to DSBs through PAR binding [54]. While vertebrate Ku70 does not contain this PBZ domain, it does raise the question of the role PAR plays in the recruitment of vertebrate Ku70 to DSBs *in vivo.* The pADPr motif of Ku70 may act to coordinate its interaction with PARylated DNA-PK as previously predicted [36], and with PARylated PARP-1 or other proteins at the DNA damage sites. Competition between Ku70 and PARP-1 has been shown most effectively *in vitro* [41], [55], but neither of these studies examined the PARylation state of PARP-1 or Ku70. Additionally, Ku represses backup-NHEJ under normal conditions [33], [34], [41] making the interplay between PARP-1, PARylation, and Ku70 an important target for further investigation.

Our findings using pol β null cells and MMS treatment initially appeared consistent with another study, which also noted an increase in PAR levels compared with wild-type cells. However, those authors attributed the increase in PAR as a cell death signal leading to PARP-1-mediated necrosis [14]. In contrast, the dramatic increase in PAR observed here in pol β null cells within 10 min after MMS exposure was down-regulated to the untreated cellular level within 30 min after exposure. In order to verify that necrotic cell death was not initiated by depletion of intracellular ATP under our treatment conditions, we measured intracellular ATP levels (Figure S1) and the fraction of necrotic cells 24 h after MMS exposure (Figure 6). These data indicated that the observed PARP-1 hyperactivation in pol β null cells did not induce necrosis and failed to deplete ATP levels.

**Figure 7:**
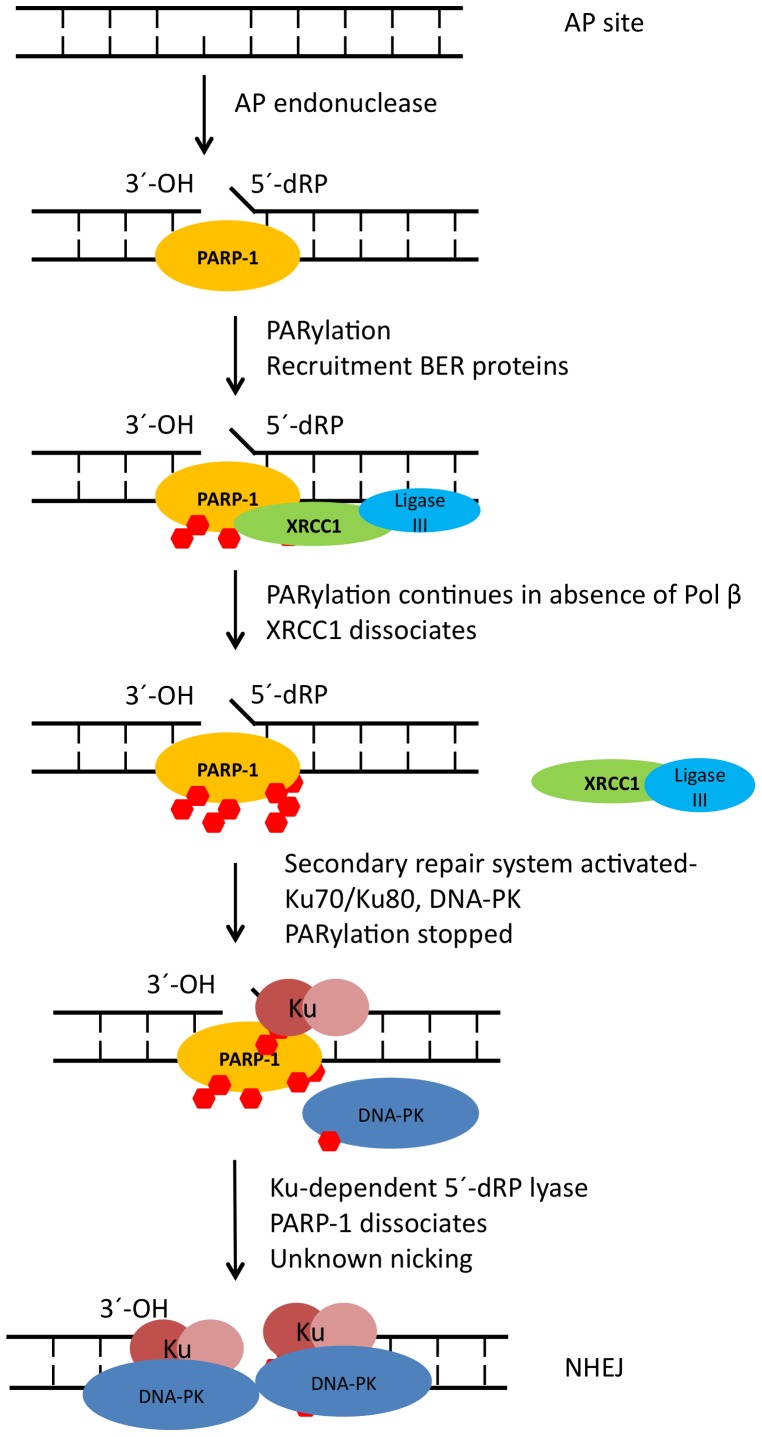
Model of PARP-1 hyperactivation and recruitment of NHEJ proteins.

Taken together, our results suggest a model where BER deficiency leads to increased production of PAR by PARP-1, and this serves to amplify a damage signal while activating and recruiting downstream repair elements to address the DNA damage (Figure 7). Whether the PARylated PARP-1 is maintained at the site of DNA damage by the toxic unrepaired BER intermediate [6] or dissociates from the DNA when a PARylation maximum is reached is unclear [56]. The decrease in PAR levels observed at 30 min, which coincides with the increasing interaction of Ku70 and PARP-1, leads us to propose that PARP-1 is likely retained at the site of damage to sequester toxic BER intermediates until significant amounts of Ku70 can be recruited. Ku70 then competes for DNA binding with PARP-1 leading to dissociation of PARP-1 from DNA. It is possible that Ku70 can utilize its dRP lyase activity [57], [58] to remove blocks to subsequent repair. How unrepaired BER intermediates are converted to DSBs is still unknown at this point. Possibly the extended remodeling of chromatin by PARP-1 at the sites of unrepaired damage assists this conversion. Once DSBs occur, classical-NHEJ ensues.

While we cannot exclude other DNA repair mechanisms in cells when BER is defective, our evidence points to classical-NHEJ rather than backup-NHEJ being enlisted to rescue stalled base lesion repair intermediates. This link between PARP-1 hyperactivation and hand-off to classical-NHEJ provides evidence of cross-talk between the roles of PARP-1 in BER/SSB and DSB repair.

## Supporting Information

Figure S1 **Intracellular ATP levels of wild-type and pol β null cells after exposure to MMS and given increasing amounts of time to repair.**
(TIFF)
