# Supplementary figures and images for: Hyperactivation of PARP Triggers Nonhomologous End-Joining in Repair-Deficient Mouse Fibroblasts
Source: PLoS One. 2012 Nov 7;7(11):e49301. doi: 10.1371/journal.pone.0049301 (PMC3492265; doi:10.1371/journal.pone.0049301)

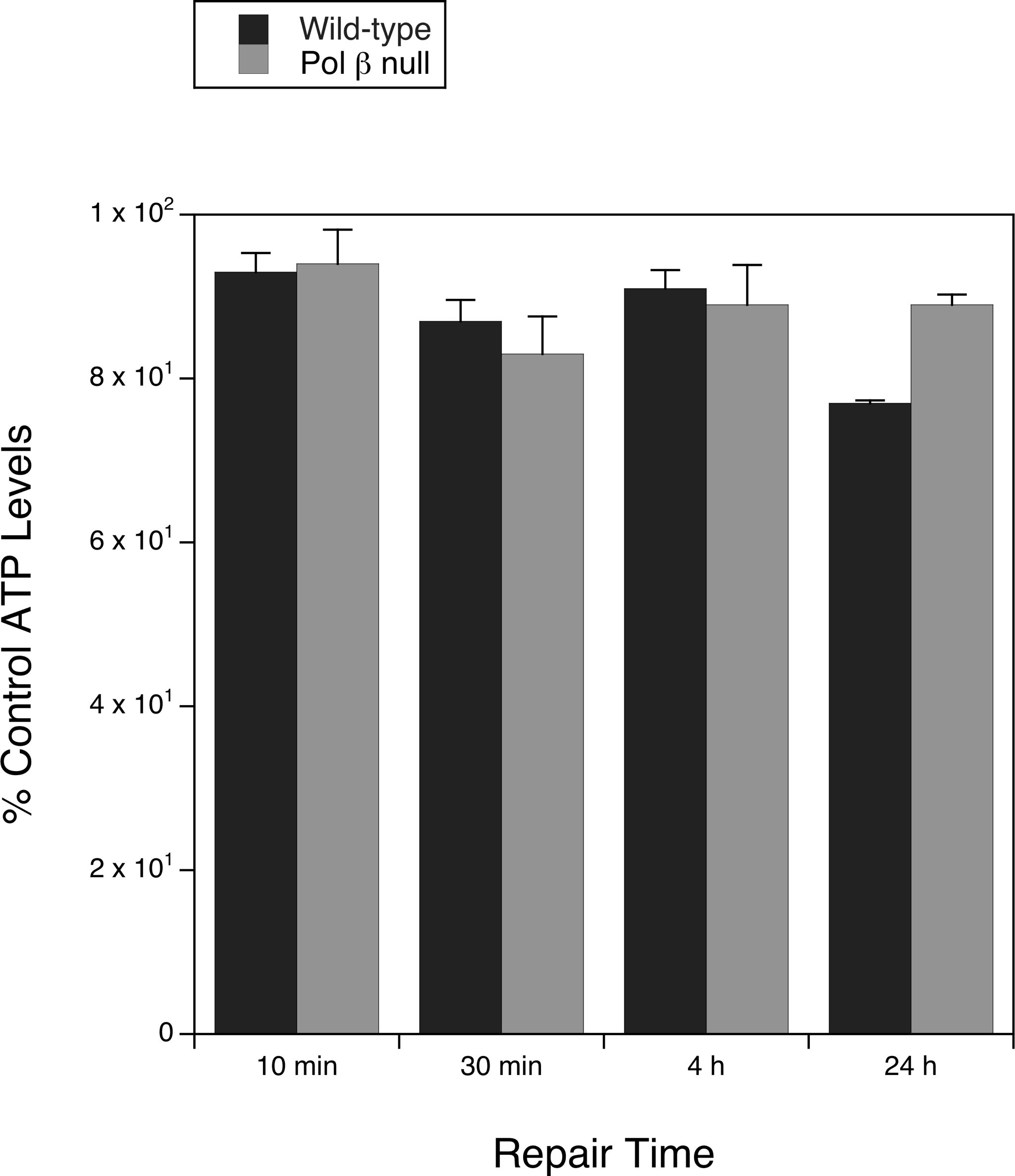

Supplement: Figure S1 — Intracellular ATP levels of wild-type and pol β null cells after exposure to MMS and given increasing amounts of time to repair. (TIFF) [file pone.0049301.s001.tif]
